# Human Cytomegalovirus Infection in Haematopoietic Stem Cell Transplant Recipients and CAR‐T Cell Recipients—*PART 2: Antiviral Therapy and Virus‐Specific T Cell Therapy for HCMV in Allo‐HSCT*

**DOI:** 10.1002/rmv.70135

**Published:** 2026-03-31

**Authors:** Gaurav Sutrave, Michelle K. Yong, Danya Kaplan, David J. Gottlieb, Allison Abendroth, Barry Slobedman, Emily Blyth, Lauren Stern

**Affiliations:** ^1^ Blood Transplant and Cell Therapies Program Department of Haematology Westmead Hospital Sydney New South Wales Australia; ^2^ Sydney Medical School Faculty of Medicine and Health The University of Sydney Sydney New South Wales Australia; ^3^ Westmead Institute for Medical Research Westmead New South Wales Australia; ^4^ Department of Infectious Diseases Peter MacCallum Cancer Centre Melbourne Victoria Australia; ^5^ Sir Peter MacCallum Department of Oncology The University of Melbourne Melbourne Victoria Australia; ^6^ Department of Infectious Diseases Royal Melbourne Hospital Melbourne Victoria Australia; ^7^ National Centre for Infections in Cancer Peter MacCallum Cancer Centre Melbourne Victoria Australia; ^8^ Infection, Immunity, and Inflammation School of Medical Sciences Faculty of Medicine and Health The University of Sydney Sydney New South Wales Australia; ^9^ Charles Perkins Centre the Sydney Institute for Infectious Diseases The University of Sydney Sydney New South Wales Australia

**Keywords:** antivirals, cytomegalovirus, haematopoietic stem cell transplant, HCMV, virus‐specific T cell, VST

## Abstract

Human cytomegalovirus (HCMV) reactivation is a common and significant complication after allogeneic haematopoietic stem cell transplantation (allo‐HSCT), causing potentially life‐threatening disease. Antiviral therapy approaches for HCMV are often limited by toxicities and the risk of developing antiviral drug resistance. This review article (Part 2) provides an overview of current antiviral pharmacotherapies for HCMV and the application of virus‐specific adoptive T cell therapies for the prevention or treatment of HCMV reactivation in allo‐HSCT recipients. The number of available antiviral drugs for HCMV is expanding, and letermovir primary prophylaxis is increasingly being adopted due to its favourable safety profile. Treatment resistant/refractory infections, end‐organ disease, and late HCMV reactivations after antiviral therapy withdrawal continue to pose challenges. Adoptive HCMV‐specific T cell therapies are a promising strategy for promoting immune‐mediated control of HCMV reactivation in allo‐HSCT recipients. The administration of HCMV‐specific T cell products, generated through ex vivo expansion of donor‐derived or partially HLA matched, third party HCMV‐specific T cells, have demonstrated efficacy in combatting clinically significant HCMV infection in clinical trials. Adoptive HCMV‐specific T cell therapies represent a powerful alternative approach for managing drug resistant HCMV infections in allo‐HSCT recipients and reducing the reliance on antiviral pharmacotherapies.

**Abbreviations**
ACT: Adoptive T cell therapy
Allo‐HSCT: Allogeneic haematopoietic stem cell transplant
csHCMVi: Clinically significant HCMV infection
D−: HCMV seronegative donor
EBV: Epstein Barr virus
gB: Glycoprotein B
GvHD: Graft‐versus‐host disease
HCMV: Human cytomegalovirus
HCMV‐VSTs: HCMV virus specific T cells
HLA: Human leucocyte antigen
PBMC: Peripheral blood mononuclear cells
R+: HCMV seropositive recipient
R−: HCMV seronegative recipient
VST: Virus specific T cell

## 1 Introduction

The betaherpesvirus human cytomegalovirus (HCMV) is an important opportunistic pathogen in allogeneic haematopoietic stem cell transplant (allo‐HSCT) recipients, responsible for morbidity and mortality (see ‘*Part 1: Risk factors, clinical impact and immune response’*, https://doi.org/10.1002/rmv.70142). HCMV reactivation and end‐organ disease after allo‐HSCT are typically managed using antiviral drug therapies in prophylactic or pre‐emptive treatment regimens, guided by regular quantitative viral surveillance in the blood. Although antivirals are often effective in both the prevention and treatment of HCMV, their use can lead to several adverse clinical events such as medication‐related organ toxicity, post‐prophylaxis HCMV recurrences and the development of drug resistance [1]. Virus‐specific adoptive T cell therapies have emerged as an alternative approach for the treatment and prevention of clinically significant HCMV infection (csHCMVi) in allo‐HSCT recipients, and can promote sustained viral control while reducing antiviral therapy usage. In this review article, we survey current antiviral therapies and the development of HCMV‐specific T cell therapies for HCMV infection in allo‐HSCT recipients.

## 2 Antiviral Treatment Approaches for HCMV Reactivation in HSCT Patients

The armamentarium of HCMV antiviral medications now includes letermovir and maribavir in addition to older agents such as (val)ganciclovir, foscarnet and cidofovir. These antivirals are the cornerstone to treating csHCMVi and disease following HSCT by directly targeting the HCMV virus and inhibiting replication [2, 3]. Antivirals can be prescribed as prophylaxis in HCMV seropositive recipients (e.g., letermovir), as pre‐emptive treatment for clinically significant HCMV infection (e.g., valganciclovir, ganciclovir or foscarnet), or in the treatment of resistant HCMV disease (e.g., maribavir). As well as having direct antiviral effects, treatment of clinically significant HCMV infection may also have beneficial indirect effects on non‐relapse mortality [4, 5, 6].

### 2.1 Letermovir

Letermovir is an oral drug inhibitor of the HCMV terminase which is now widely recommended and adopted as standard of care as prophylaxis in HCMV seropositive recipients of allo‐HSCT [7, 8]. Currently, however, the adoption and use of letermovir prophylaxis varies in different geographic regions, with access limited by financial barriers in some countries (e.g. Australia, where letermovir is not subsidised [9]). Prophylaxis with letermovir has significantly changed the landscape of post‐transplant HCMV infection with HCMV seropositive recipients now experiencing reduction in the incidence of early csHCMVi, reduction in the incidence of end‐organ disease, reduced resistant refractory HCMV and improved survival outcomes [4, 10]. In the seminal Phase 3 randomised letermovir trial, where letermovir prophylaxis was administered until week 14 post‐transplant, all cause mortality by week 24 post‐transplant was significantly reduced in letermovir recipients compared to placebo [6]. The survival disadvantage observed in HCMV seropositive (R+) recipients compared with D‐/R‐, was recently shown to be mitigated by the use of letermovir prophylaxis, suggesting that poor transplant outcomes previously observed in these patients could be attributable to HCMV reactivation [11].

There was not a significant difference in the incidence of all cause mortality at week 48 post‐transplant between letermovir recipients and placebo recipients in the original Phase 3 letermovir trial [6]. Late onset HCMV infection is often observed following cessation of letermovir prophylaxis in approximately 12%–45% of patients [12]. Following this observation, a Phase 3 randomised placebo‐controlled clinical trial of extended duration letermovir prophylaxis trial was performed where high risk recipients were randomised to 200 days of letermovir or placebo. The trial demonstrated that letermovir prophylaxis reduced the risk of csHCMVi to day 200, but the overall incidence of csHCMVi was similar by week 48 [13]. High risk patients who would benefit include patients who received allografts from a mismatched or haploidentical donor, umbilical cord transplant, undergoing T cell depletion, or received systemic prednisone greater than 1 mg/kg/day within 6 weeks of day 100 after HSCT [7, 13].

Letermovir has a unique mechanism of action which limits cross resistance to other antivirals [14]. However, the drug has a low barrier to resistance particularly in the presence of active HCMV replication and thus should not be prescribed in the treatment of resistant, refractory HCMV or outside of prophylaxis [2, 15]. Recent studies have also shown letermovir to be effective in secondary prophylaxis [16, 17, 18]. During letermovir prophylaxis, clinicians often observe small viral load rises which are less likely to represent genotype proven letermovir drug resistance [19], but more commonly are low‐level self‐resolving HCMV DNAemia episodes or isolated HCMV DNA ‘blips’ which do not require treatment interruption [8, 20].

The rebound in csHCMVi events after letermovir prophylaxis cessation suggests that HCMV‐specific immune reconstitution also plays an important role in post‐transplant survival, rather than simply suppression of HCMV replication alone. Interesting, a lower incidence of all cause mortality at week 48 post‐transplant in letermovir recipients with csHCMVi (15.8%) compared to placebo recipients with csHCMVi (31.0%) was noted in a mortality analysis of the original Phase 3 letermovir trial [5], warranting further investigation into the mechanisms by which letermovir prophylaxis may alter clinical outcomes.

### 2.2 Maribavir

Maribavir is an oral anti‐HCMV agent recommended in the treatment of resistant and refractory HCMV infection or disease following transplantation [7, 8, 21]. In a Phase 3 study of refractory and/or resistant HCMV infections, a significantly higher percentage of patients randomised to maribavir compared to physician assigned treatment achieved viral suppression after 8 weeks [21]. Additionally, maribavir treated patients experienced less side effects such as neutropenia or renal impairment and were more likely to complete a course of treatment compared to ganciclovir or foscarnet [21]. It is important to note that maribavir is not recommended to treat central nervous system or ocular HCMV disease due to poor ability to cross the blood‐brain barrier, and like letermovir, valaciclovir prescribing is required to cover other herpes viruses [2]. Real world studies of maribavir in resistant refractory HCMV infection have shown similar efficacy results to the trial—although, often in combination with other HCMV therapies [22].

Maribavir is a competitive inhibitor of the HCMV serine/threonine protein kinase UL97, and inhibits HCMV DNA replication and interferes with viral encapsidation and nuclear egress [14]. Co‐administration of valganciclovir or ganciclovir with maribavir is not recommended as the activity of ganciclovir is dependent on phosphorylation by the HCMV UL97 kinase [23]. Drug resistance to maribavir at UL97 has been reported in both clinical trials and real‐world use and thus is an important clinical issue to closely monitor as maribavir prescribing expands over the coming years [15, 24]. Maribavir resistance has been found to emerge earlier and more frequently than valganciclovir resistance [25], suggesting that viral genotypic testing should be performed early if there is evidence of lack of response to maribavir.

### 2.3 Valganciclovir, Ganciclovir And/Or Foscarnet

Valganciclovir, ganciclovir and/or foscarnet currently remain as the first line anti‐HCMV agents in the pre‐emptive treatment of csHCMVi [3, 8]. In a recent Phase 3 double blind randomised study of treatment for asymptomatic first HCMV infection post allo‐HSCT, participants were randomised to either maribavir or valganciclovir [26]. Maribavir did not achieve non‐inferiority compared to valganciclovir (69.6% vs. 77.4%) where the primary endpoint was HCMV clearance at week 8 (pre‐specified 7% non‐inferiority). Key secondary endpoints were achieved such as HCMV clearance at week 16 and safety outcomes showing neutropenia was much less common with maribavir than valganciclovir (16.1% vs. 54.2%). Fewer patient discontinuations occurred with maribavir than valganciclovir due to adverse events (27.8% vs. 41.2%) demonstrating better tolerability [26].

## 3 Adoptive T Cell Therapy for HCMV

Improvements in the available diagnostics for the detection of HCMV, as well as the expanding array of HCMV specific pharmacotherapies has resulted in a gradual reduction in the incidence of HCMV‐related mortality in immunosuppressed individuals, including allo‐HSCT recipients. However, currently available HCMV‐specific pharmacotherapies are limited by medication related adverse effects, the development of drug resistance and the need for prolonged use [1]. Most critically, none of the currently available pharmacotherapies are capable of enhancing the HCMV‐specific immune responses which are most critical for long term viral control [27]. Thus, many of these patients suffer from recurrent or relapsing infections upon withdrawal of pharmacotherapy, exposing them to further risks of long term antiviral therapy [6].

Adoptive T cell therapy (ACT) has emerged as an attractive therapeutic strategy for csHCMVi and potentially for prophylaxis and pre‐emptive HCMV treatment. First described by Riddell and colleagues [28], this process involves the ex vivo isolation and/or expansion of HCMV virus‐specific T cells (HCMV‐VSTs) derived from healthy haematopoietic stem cell donors, which are then infused into patients with csHCMVi [29]. The infused cells are capable of simultaneously clearing active virus while also establishing long term HCMV‐specific immune responses, thereby preventing recurrent or relapsing infection [30].

**FIGURE 1:**
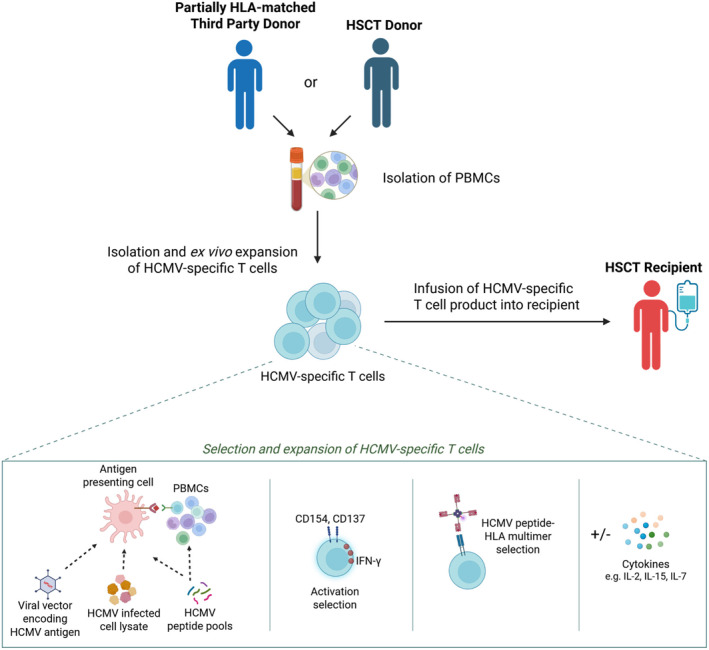
Expansion and isolation of HCMV‐specific T cells for adoptive cell therapy in allo‐HSCT. Human cytomegalovirus (HCMV)‐specific T cell products are generated from peripheral blood mononuclear cells (PBMCs) obtained from a healthy partially HLA‐matched third‐party donor or the allogeneic haematopoietic stem cell transplant (HSCT) donor. Virus‐specific T cells are then selectively isolated and expanded ex vivo using a variety of methods. HCMV‐specific T cells can be selectively expanded from PBMCs ex vivo through co‐culture with antigen presenting cells (e.g., monocyte‐derived dendritic cells) that present processed HCMV peptides (e.g., through pulsing with viral peptide pools or via infection with a viral vector encoding HCMV antigens). HCMV‐specific T cells can be also be enriched preceding ex vivo expansion by sorting (e.g., using fluorescence‐activated cell sorting or immunomagnetic bead selection) by selecting on the expression of activation markers such as CD137 or CD154 following pulsing with HCMV peptide pools. Alternatively HCMV‐specific T cells can be directly isolated for infusion by selection based on expression of the cytokine interferon gamma (IFN‐γ) after exposure to HCMV peptide pools, or by binding of HCMV peptide‐HLA multimers to virus‐specific T cells. Isolated HCMV‐specific T cells may be stimulated with cytokines to promote expansion. IL (interleukin), human leucocyte antigen (HLA). Created in BioRender. Stern, L. (2026) https://BioRender.com/eb1la9e.

These HCMV‐VST products are generated through the selective isolation and expansion of HCMV‐specific T cell clones from peripheral blood mononuclear cells (PBMC) acquired from a healthy donor (Figure 1). The initial manufacturing protocols for HCMV‐VSTs relied upon repetitive cycles of stimulation of the starting PBMCs with appropriately HLA matched HCMV‐infected fibroblasts or antigen presenting cells. This process was time consuming and the infused HCMV‐VSTs had limited proliferative capacity and poor persistence [31]. Subsequent iterations of HCMV‐VST manufacture have thus focused on improving initial HCMV specific T cell isolation and reducing the number of ex vivo stimulations to achieve adequate T cell expansion and avoid T cell exhaustion. Advances in the understanding of the HCMV‐specific immunodominant peptides and their HLA restriction has allowed for more efficient HCMV‐specific T cell isolation from PBMCs [32], and their selective ex vivo expansion has been achieved through avoiding the necessity to generate antigen presenting cells [33, 34], optimising T cell stimulatory cytokine cocktails and improving culture flask technology [35].

### 3.1 Donor Derived HCMV‐VSTs

Several studies have now been conducted to evaluate the safety and efficacy of HCMV‐VST infusions to treat csHCMVi in immunosuppressed individuals, most commonly following allo‐HSCT. The initial studies were conducted using HCMV‐VST products manufactured from the original HSCT donor. The fully HLA matched nature of these infused HCMV‐VST products reduced the risk of rejection of the infused product via recipient derived anti‐HLA antibody mediated responses, as well as the theoretical risk of graft‐versus‐host disease (GvHD) mediated by alloreactive T cells within the infused HCMV‐VST product. When used to treat HCMV infection post‐HSCT, these donor derived HCMV‐VSTs demonstrated response rates of 66%–100% (the majority of which were complete responses). The infused HCMV‐VSTs expand in vivo leading to viral clearance [36, 37], while gene marking studies have demonstrated that infused T cells can persist for up to 9 years [38] providing long term immunological surveillance. There was minimal infusion related toxicity and no increase in GvHD over untreated contemporaneous control populations (Table 1). HCMV‐VSTs have also been utilised for prophylactic and pre‐emptive therapy and have demonstrated efficacy in minimising the rates of HCMV viraemia and infection and antiviral pharmacotherapy use [29, 31, 33, 39, 40].

**TABLE 1:** Selected clinical trials of donor derived HCMV‐VST therapy for clinically significant HCMV post‐HSCT (adapted from Sutrave et al. [63]).

Study (reference)	Patients enroled	Clinical efficacy	Safety
Einsele et al. (2002) [64]	8 Patients	CR in 6/8 (5 required 1 infusion, 1 required 2 infusions) PR in 1/8 NE in 1/8	No GvHD
Peggs et al. (2003) [31]	16 Patients	CR in 8/16	3/16 grade I skin aGvHD
Cobbold et al. (2005) [65]	9 Patients	CR in 8/9 PR in 1/9	1/9 grade I aGvHD 1/9 grade II aGvHD
Feuchtinger et al. (2010) [32]	18 Patients	CR/PR in 15/18 PD in 3/18	No GvHD
Dong et al. (2010) [66]	2 Patients	CR in 2/2	No GvHD
Schmitt et al. (2011) [67]	2 Patients	CR in 2/2	No GvHD
Peggs et al. (2011) [40]	11 Patients	CR in 11/11	3/11 grade II‐III aGvHD 1/11 cGvHD
Uhlin et al. (2012) [68]	2 Patients	CR in 2/2	No GvHD
Meij et al. (2012) [69]	6 Patients	CR in 6/6	No GvHD
Bao et al. (2012) [70]	7 Patients	CR in 6/7 NR in 1/7	No GvHD
Koehne et al. (2015) [71]	16 Patients	CR in 14/16 PR in 1/16 NR in 1/16	No GvHD
Neuenhahn et al. (2017) [72]	9 Patients	CR in 5/9 PR in 2/9 NR in 1/9 NE in 1/9	1/9 grade III gut aGvHD
Pei et al. (2017) [73]	32 Patients	CR in 27/32 NR in 5/32	1/32 grade II aGvHD
Abraham et al. (2019) [74]	4 Patients	CR in 4/4	1/4 grade III aGvHD
Fabrizio et al. (2021) [52]	25 Patients	CR/PR in 17/25 SD/PR in 4/25 NE in 4/25	Not reported
Pei et al. (2022) [75]	190 Patients	CR in 170/190	28/190 grade I‐II aGvHD
Wang et al. (2022) [76]	10 Patients	CR/PR in 10/10	No GvHD
Ruan et al. (2022) [77]	19 Patients	CR/PR in 17/19	No GvHD
Heinz et al. (2023) [78]	15 Patients	CR/PR in 15/15	No GvHD
Jiang et al. (2024) [79]	40 Patients	CR/PR 26/40	4/40 grade I‐II aGvHD

Abbreviations: aGvHD, acute graft‐versus‐host disease; cGvHD, chronic graft‐versus‐host disease; CR, complete response; GvHD, graft‐versus‐host disease; NE, not evaluable; NR, no response; PD, progressive disease; PR, partial response; SD, stable disease.

Despite their clinical efficacy, the more widespread adoption of donor derived HCMV‐VST therapy has been limited by their bespoke nature (being patient specific and donor derived) with reasonably prolonged manufacturing time when taking donor clearance, starting PBMC collection and quality control into account. Furthermore, while HCMV‐VST products are readily manufactured from seropositive donors, in the case of seronegative or umbilical cord donors, repeated antigenic stimulation and additional manufacturing steps are required to generate HCMV‐VST products [41]. This results in higher costs, longer time to treatment and inability to have a product available at the time of clinical need. This is of particular relevance in the post‐HSCT period, where HCMV can cause rapidly progressive infections which can preclude the administration of donor derived HCMV‐VST products if manufacturing is only initiated at the time of infection diagnosis or relapse.

### 3.2 Partially HLA Matched, Third Party HCMV‐VSTs

In order to address these limitations, the use of cryopreserved banks of HCMV‐VST products covering the most common HLA molecules within the population have been explored as an alternative modality to treat csHCMVi following HSCT. The development of these banks has arisen from the recognition that HCMV‐VST products need only share a single HLA through which immunodominant HCMV peptides are presented with the intended recipient. The choice of donors with HLA molecules common in the intended population for treatment allows for the creation of a suite of ‘off‐the‐shelf’ products from a limited number of donors that can utilised at the time of clinical need [42]. These banked, partially HLA matched third party HCMV‐VST products have demonstrated near equivalent efficacy for the treatment of clinically significant HCMV infections in the post‐HSCT period, with response rates of 75%–100% (Table 2). Furthermore, treatment of HCMV post‐HSCT with third party HCMV‐VST appears to be associated with reduced total duration of antiviral pharmacotherapy and improved overall survival compared to historical controls [43, 44].

**TABLE 2:** Selected clinical trials of partially HLA matched, third party HCMV‐VST therapy for clinically significant HCMV post‐HSCT (adapted from Sutrave et al. [63]).

Study (reference)	Patients enroled	Clinical efficacy	Safety
Uhlin et al. (2012) [68]	4 Patients	CR in 2/4 PR in 1/4 SD in 1/4	No GvHD
Leen et al. (2013) [48]	23 Patients	CR in 9/23 PR in 8/23 SD/PD in 2/23 NE in 4/23	2/23 grade I aGvHD 1/23 grade II aGvHD
Koehne et al. (2015) [71]	1 Patient	CR in 1/1	No GvHD
Naik et al. (2016) [80]	7 Patients	CR/PR in 6/7	2/7 with grade II aGvHD 1/7 with cGvHD
Withers et al. (2017) [81]	28 Patients	CR in 22/28 PR in 5/28 SD in 1/28	2/28 aGvHD 5/28 cGvHD
Kallay et al. (2018) [82]	6 Patients	CR/PR in 6/6	No GvHD
Jiang et al. (2022) [44]	27 Patients	CR in 25/27 PR in 2/27	1/27 grade II aGvHD 3/27 grade III‐IV aGvHD 7/27 with cGvHD
Ruan et al. (2022) [77]	10 Patients	CR/PR in 9/10	No GvHD
Prockop et al. (2023) [83]	59 Patients	CR in 20/59 PR in 18/59 SD/PD in 21/59	1/59 grade III aGvHD
Pfeiffer et al. (2023)^a^ [84]	24 Patients	CR in 11/24 PR in 12/24	No grade III‐IV aGvHD
Jiang et al. (2024) [79]	13 Patients	CR/PR in 9/13	2/13 grade I‐II aGvHD
Keller et al. (2024)^a^ [45]	20 Patients	CR/PR in 10/20 SD/PD in 3/20 NE in 7/20	1/20 aGvHD
Neller et al. (2024)^a^ [85]	20 Patients	CR/PR in 12/20	Not reported

Abbreviations: aGvHD, acute graft‐versus‐host disease; cGvHD, chronic graft‐versus‐host disease; CR, complete response; GvHD, graft‐versus‐host disease; NE, not evaluable; PR, partial response; PD, progressive disease; SD, stable disease.

^a^ Multivirus specific T cell product. Outcomes for patients treated for HCMV only reported.

Despite the HLA mismatch, third party HCMV‐VSTs have been generally well tolerated with no demonstrated increase in the incidence of GvHD or infusion related toxicity compared to fully HLA matched, donor‐derived HCMV‐VSTs. Rare toxicities have been more recently described following third party VST infusion, usually in the context of an infusional product targeting multiple viruses simultaneously (including HCMV). These include a case of cytokine release syndrome in a recipient with disseminated adenovirus [45], and another patient with secondary graft failure with associated VST expansion (although a specific alloreactive clone could not be isolated) [46].

Following infusion, third party HCMV‐VSTs initially expand in vivo in a similar manner to fully HLA matched, donor derived HCMV‐VSTs, however the third party HCMV‐VSTs do not appear to persist in circulation beyond 120 days [44, 47, 48]. In spite of this, the pattern of initial rapid viral control by 3–4 weeks post‐infusion, followed by long term remission mirrors that seen with donor derived HCMV‐VSTs which persist for much longer [49]. This pattern of long term viral control without persistence of infused virus‐specific T cells is also seen in patients treated with partially HLA matched third party Epstein Barr virus (EBV) specific T cells [50, 51]. This suggests that the infused HCMV‐VSTs stimulate reactivation of a recipient‐derived HCMV‐specific T cell response, which then mediates long term viral control after attrition of the infused HCMV‐VSTs. This is further supported by higher rates of virologic response to HCMV‐VST infusion in recipients with higher pre‐infusion CD4^+^ T cells [52]. This process, known as epitope spreading, has been described following infusion of autologous EBV‐specific T cells in patients with EBV positive malignancies and may underpin the clinical efficacy of partially HLA matched third party HCMV‐VSTs [53].

### 3.3 Limitations of HCMV‐VST Therapy

In spite of their clinical efficacy, HCMV‐VSTs have a few demonstrated limitations to their broader application across all populations. Most clinical trials have excluded patients on high dose corticosteroids (> 1 mg/kg prednisone or equivalent), within 4 weeks of T cell lympholytic therapy (such as alemtuzumab or anti‐thymocyte globulin) or with active GvHD post‐HSCT, all of which are populations not only at a high risk of viral reactivation but also less likely to have optimal responses to HCMV‐VSTs [45]. The clinical efficacy of HCMV‐VSTs relies on at least one shared HLA between the infused product and the recipient, the shared allele should be the one through which virus specific activity is mediated [45]. In the case of rare HLA alleles are present, no product may be available thus limiting the availability of this therapy for all patients. Implementation of processes to actively recruit donors with less common HLA alleles (e.g., from immigrant and Aboriginal and Torres Strait Islander communities) could broaden the coverage and enhance the clinical applicability of HCMV‐VST cell banks. Lastly, the utilisation of HCMV‐VST therapy requires local clinical and laboratory expertise to ensure appropriate administration and clinical monitoring. The infrastructure for manufacture of VST products is a significant investment to establish and maintain and commercial development of VSTs has had limited success. Tablecleucel, an allogeneic, banked EBV VST product, is authorised for treatment of EBV‐related post‐transplant lymphoproliferative disease in the EU but was rejected by the FDA in January 2026. There are no commercially available HCMV VST products. Access to allogeneic VSTs via centralised cell banks that cover the local HLA diversity, along with a framework for distribution and reimbursement may improve access.

The limited persistence of third party HCMV‐VSTs also raises the role for repeat infusions to deepen response, with second and later infusions demonstrating response rates of 22%–100% [44, 45]. Finally, the heterogeneity of the starting PBMCs and manufacturing protocols for HCMV‐VST product manufacture results in significant variability in the final HCMV‐VST product characteristics. As a result, establishing the therapeutic index and optimal dose of HCMV‐VSTs to achieve viral clearance has been difficult to determine. While some studies have shown no difference in virologic outcome regardless of whether a pre‐specified VST dose is administered [43], there is no universally accepted optimal HCMV‐VST infusion composition (in terms of CD4:CD8 ratio or T cell immunophenotype) to maximise therapeutic efficacy. While several Phase 1/2 studies have demonstrated safety of HCMV‐VSTs, thus far there have been no randomised studies demonstrating superiority of HCMV‐VSTs over currently available pharmacotherapy. Further studies are still required in order to determine the optimal dose and frequency of HCMV‐VSTs to maximise efficacy, minimise toxicity and thus potentially provide additional benefit over anti‐HCMV drug therapies.

Ultimately, HCMV‐VSTs appear to be an attractive ACT particularly for drug resistant infections given that there is no current evidence that anti‐HCMV drug resistance confers cross‐resistance to HCMV‐VSTs. This has resulted in their incorporation into current HCMV management guidelines [8, 54]. The role of HCMV‐VSTs in earlier lines of therapy (e.g., at first viral reactivation or as prophylaxis) and advantages over the currently available pharmacotherapies in this context are areas of ongoing investigation which will assist in determining the precise positioning of this therapeutic modality in the HCMV treatment algorithm.

## 4 Vaccination for HCMV

Although numerous vaccine candidates have been developed and many have advanced to clinical trials [55], there is currently no licenced vaccine for HCMV. Vaccination could be employed as a prophylactic or therapeutic strategy in allo‐HSCT to stimulate antiviral immune responses in the recipient, and could also be applied to boost HCMV‐specific immunity in the donor [56]. HCMV vaccines under current evaluation include glycoprotein B (gB) subunit, attenuated viral vector, and mRNA‐based vaccines [55, 57]. The Triplex vaccine is a modified vaccinia Ankara (MVA) vector based vaccine designed to accelerate the recovery of HCMV‐specific T cell immunity and encodes three immunodominant HCMV antigens (pp65, IE1‐exon4 and IE2‐exon5) [58]. In a Phase 2 trial, HCMV seropositive recipients vaccinated with Triplex at days 28 and 56 post‐HSCT exhibited increased HCMV pp65‐specific T cell responses and had a lower incidence of HCMV reactivation by day 100 post‐transplant, compared to placebo [59]. The Triplex vaccine is now being evaluated in a Phase 2 trial to determine whether vaccination of HLA‐matched related donors enhances HCMV‐specific immunity and prevents csHCMVi in HSCT recipients (NCT06059391).

An mRNA‐based vaccine, mRNA‐1647 (Moderna), which encodes HCMV gB and pentameric complex [60], failed to demonstrate benefit compared to placebo in the prevention of primary HCMV infection in healthy seronegative females of childbearing age in a Phase 3 clinical trial (NCT05085366). The utility of this product is being evaluated in a Phase 2 clinical trial of prevention of csHCMVi following the cessation of HCMV antiviral prophylaxis following allo‐HSCT (NCT05683457). Other HCMV vaccine candidates previously tested in allo‐HSCT recipients such as PepVax (chimaeric peptide‐based; Phase 2 trial) [61] and ASP0113 (DNA‐based; Phase 3 trial) [62] did not meet efficacy endpoints in reducing HCMV viraemia or end‐organ disease. Ongoing development of effective vaccines to stimulate protective HCMV‐specific immune responses in allo‐HSCT recipients is needed as vaccination would serve as a valuable tool for preventing and managing csHCMVi in transplant patients.

## 5 Conclusion

Continued advances in the prevention and treatment of opportunistic HCMV reactivation and disease in transplant recipients offer a pathway towards reducing the morbidity and mortality caused by HCMV. Although the repertoire of antiviral therapies available or under development is expanding, there are a number of remaining challenges, including potential toxicities, antiviral resistance, treatment refractory infections, financial costs and equitable access to new and emerging therapies. Defining the optimal HCMV management strategy for each patient and among at risk groups at a broader level will require greater understanding of the key risk profiles for HCMV complications and responses to antiviral therapy, as well as the optimal timing and selection of different therapy types, depending on viral and host parameters. Finally, it is important to consider how antiviral interventions, such as drug therapies and ACTs, intersect with the landscape of post‐transplant immune reconstitution, in particular the recovery of HCMV‐specific cell‐mediated immunity, which is vital for sustained viral control. Further development and evaluation of immune monitoring strategies to tailor antiviral treatment administration for individual patients will also lead to better treatment outcomes for HCMV in allo‐HSCT recipients in the future.

## Author Contributions

L.S., A.A., B.S., and E.B. conceptualised the manuscript. G.S., M.K.Y., and L.S. assembled the initial draft. All authors reviewed and approved the manuscript.

## Funding

This work was supported by NHMRC Ideas Grant 2037493 awarded to BS and EB and NHMRC Ideas Grant 2019871 awarded to BS and AA, and a Translational Programme Grant from the Cancer Council NSW, a grant from the Leukaemia Foundation of Australia and a Centre for Research Excellence grant from the NHMRC awarded to DJG.

## Conflicts of Interest

E.B. holds patents in adoptive cell therapy for opportunistic infection and malignancy. E.B. reports advisory board membership for IQVIA, AbbVie, MSD, Astellas, Novartis, Gilead, Adcella and Bristol Myers Squibb, and research funding from MSD.

## Data Availability

The data that support the findings of this study are available from the corresponding author upon reasonable request.
